# TSH and Thyrotropic Agonists: Key Actors in Thyroid Homeostasis

**DOI:** 10.1155/2012/351864

**Published:** 2012-12-30

**Authors:** Johannes W. Dietrich, Gabi Landgrafe, Elisavet H. Fotiadou

**Affiliations:** ^1^Lab XU44, Medical Hospital I, Bergmannsheil University Hospitals, Ruhr University of Bochum (UK RUB), Bürkle-de-la-Camp-Platz 1, 44789 Bochum, NRW, Germany; ^2^Klinik für Allgemein- und Visceralchirurgie, Agaplesion Bethesda Krankenhaus Wuppertal gGmbH, Hainstraße 35, 42109 Wuppertal, NRW, Germany

## Abstract

This paper provides the reader with an overview of our current knowledge of hypothalamic-pituitary-thyroid feedback from a cybernetic standpoint. Over the past decades we have gained a plethora of information from biochemical, clinical, and epidemiological investigation, especially on the role of TSH and other thyrotropic agonists as critical components of this complex relationship. Integrating these data into a systems perspective delivers new insights into static and dynamic behaviour of thyroid homeostasis. Explicit usage of this information with mathematical methods promises to deliver a better understanding of thyrotropic feedback control and new options for personalised diagnosis of thyroid dysfunction and targeted therapy, also by permitting a new perspective on the conundrum of the TSH reference range.

## 1. Introduction

As thyroid hormones play a critical role for metabolism, growth, and tissue differentiation, exact and robust regulation of hormone levels is required. Although a thyrotropic hormone from anterior pituitary has first been described at the beginning of the 20th century [1, 2], it was not before 1940 that Salter postulated the existence of a control loop linking the pituitary and thyroid gland [3]. This idea was inspired by the then recent description of the two gonadotropic feedback control loops [4–7]. Only a few years later, Astwood and Hoskins independently could demonstrate both existence and pathophysiological relevance of this thyrotropic feedback control system [8–10].

Apart from a deeper insight into fundamental physiological principles, both diagnostic evaluation and dosage of substitutive therapy benefit from this knowledge. However, growing complexities of the respective relations increasingly question the validity of predictions that try to map reactions of the feedback loop to certain parameter changes. Additionally, there is an increasing gap between molecular and systems-level insights and a similar hiatus between findings of basic research and clinical applications.

Systems theoretic models try to incorporate both data from a molecular level and those from a systemic perspective on the level of the whole organism in an integrative way. Depending on their design principles the resulting cybernetic models may facilitate medical decision making and deliver hypotheses that may again serve as a basis for ongoing research.

## 2. Physiology of Thyrotropic Feedback Control

From a systems biologic perspective, thyroid homeostasis is a processing structure whose signalling is implemented by two different mechanisms, conversion and relaying [11]. Examples of relaying are the control of T4 secretion by TSH or of TSH secretion by TRH. Central and peripheral deiodinases convert T4 to the active hormone T3 and further to inactive iodothyronines. Another example of conversion processes is transport of thyroid hormones by plasma and transmembrane transporters.

### 2.1. Classical Pituitary-Thyroid Axis (Astwood-Hoskins Loop)

Apart from pituitary and thyroid, key components of the classical feedback control loop are the hypothalamus, and other organs like liver, brown adipose tissue, skeletal muscle and kidney that are capable of deiodination, as well as peripheral and central compartments, where iodothyronines distribute, act and are catabolised [12, 13]. Plasma transporters like TBG and membrane transporters like MCT8 facilitate convey of thyroid hormones in body fluids and through membranes and the blood-brain barrier [14–18].

Due to the long half-life of iodothyronines the reaction of the thyroid to stimulating TSH pulses from the pituitary is slow. A large portion of thyroxine binds reversibly to plasma proteins. Only a small free fraction (0.02% to 0.03%) is available for conversion to T3 and transport to cytoplasm. T3 is formed from T4 by 5′ deiodination at the outer ring by type 1 deiodinase predominantly in liver, kidney, and thyroid. Type 2 deiodinase mediates intracellular deiodination in glial cells, pituitary, brown adipose tissue, skeletal muscle, and placenta [19]. Obviously, intracellular deiodination facilitates feedback at the pituitary level by providing a mainly T4-dependent mechanism, which is faster than one that would depend on T3 from systemic circulation [20]. In addition, T3 is regulated by nonthyroidal factors, first of all peripheral deiodination [19, 21–25] that is subject of multiple metabolic control inputs [19, 26–30], which would also render a primarily T3-dependent feedback mechanism ineffective. High pituitary DIO2 expression rate ensures operative feedback despite T4-induced ubiquitination of type 2 deiodinase [31].

TSH is secreted in a pulsatile manner [32] with a mean pulse amplitude of 0.6 mU/L and a frequency of 5 to 20 per 24 hours [33]. Experiments suggested that there is no correlation among pulsatile secretion of TRH and TSH [34].

TSH pulses are superimposed by a 24 hour rhythm that leads to maximum TSH secretion shortly after midnight [35]. Interestingly, the interaction seems to be more than pure addition as the amplitude of short TSH pulses also rises in the second half of night. Therefore, unlike the frequency of fast pulses, their amplitude and that of diurnal rhythm of TSH seem to be controlled by TRH, as demonstrated in rat hypothalamic slices [36].

### 2.2. Ultrashort-Loop Control of Thyrotropin Incretion (Brokken-Wiersinga-Prummel Loop)

Patients suffering from Graves' disease may continue to show decreased TSH levels despite normal or even low FT4 and FT3 levels and despite being clinically euthyroid over long time periods [37, 38]. A similar constellation was described in patients with both familiar [39] and sporadic [40] activating TSH receptor mutations and in an infant born to a mother with Graves' disease [41]. This and the fact that ultrashort loop feedback control of thyrotropin secretion had been observed in rabbits [42, 43] led to the discovery of TSH receptors on folliculostellate cells of anterior pituitary lobe [44–46] and consecutive confirmation of a similar autocrine or paracrine effect in humans [47, 48]. This feedback loop might prevent excessively high TSH levels and also be a source of TSH pulsatility, as suggested by investigations based on fractal geometry [49]. 

The existence of this loop may be a challenge for interpretation of laboratory results, especially in patients with Graves' disease, where TRAbs may suppress TSH secretion independently from current FT4 levels [50] resulting in TSH levels being lower than expected in relation to current FT4 levels (see Section 4.3).

### 2.3. Long Feedback Control (Fekete-Lechan Loop)

As early as 1969 DiStefano postulated the existence of two-loop feedback of thyroid hormones targeting both hypothalamus and pituitary [51]. Based on control theoretical considerations, he had concluded that a model including proportional feedback at the hypothalamic level and rate (differential) feedback at the level of the pituitary provides best performance. At this time, unidirectional signalling from hypothalamus to pituitary resulting in stimulation of thyroid output had been described [52–54], but the existence of a long feedback loop was yet to be confirmed by experimental methods.

The presence of this additional long feedback loop that links iodothyronine levels in CNS with TRH release could be later confirmed in animal experiments [55–60]. 

Due to limitations in research methods this relationship cannot be directly investigated in humans. However, observations in animal models with induced nonthyroidal illness syndrome and phenomenologically similar observations in critically ill humans suggest this feedback loop is also effective in human physiology [61]. With current methodology the relative contribution of direct inhibition of TSH release by iodothyronine feedback and of indirect TSH reduction by suppressed TRH signalling cannot be isolated. More and more hints, however, indicate a central role of the TRH neuron in energy homeostasis, where thyroid signalling is a critical component [62, 63].

### 2.4. Alternative Mechanisms of Thyroid Control

The mentioned classical feedback mechanism controls the level of thyroid hormones via T4 formation and release. Additional mechanisms of homeostasis include autoregulation, where clearance of iodothyronines increases with their plasma levels [64–66], increased degradation of TSH in hyperthyroidism [67], possible ultrashort feedback control of TRH secretion, [68] and numerous mechanisms involving control of thyroid hormone transporters and receptor density [15, 17, 69–73]. Moreover, iodothyronines are subject to enterohepatic circulation that is a target of additional control signals [16, 74] and due to the prokinetic effects of iodothyronines possibly including thyroid hormones themselves [75, 76].

Ultrashort-loop feedback control mechanism at the site of the thyroid may exist in form of direct inhibition of TSH signalling by high levels of thyroid hormones [77–80], but from current scientific knowledge it is unclear if such a mechanism exists in humans.

Posttranslational modifications of TSH may be a possible important, but still understudied mechanism of auxiliary thyrotropic control. Like other glycoprotein hormones TSH contains asparagine-linked biantennary and triantennary oligosaccharide structures with a terminal N-acetylgalactosamine (GalNAc) sulfate signal and varying sialic acid content [81–83]. Plasma half-life of sialylated TSH is markedly prolonged, whereas asialo-TSH with terminal mannose, galactose, GalNAc sulfate, N-acetylglucosamine or fucose moieties is rapidly captured by hepatocyte asialoglycoprotein receptors and, in consequence, subject to degradation [82]. However, bioactivity of sialylated TSH seems to be reduced [84, 85]. This could be related to prolonged half-life and resulting desensitization of TSH receptors [86] by virtue of reduced TSH pulsatility. Different TSH glycoforms may contribute to overall control of thyroid homeostasis, as suggested by increased sialo-TSH content in hypothyroidism [87, 88] and decreased sialylation in nonthyroidal illness syndrome [89]. Glycosylation patterns of TSH may also be one of the reasons for concentration-independent modulation of TSH bioaction, as represented by a lack of correlation between TSH levels and FT4 concentration in central hypothyroidism [90].

In addition to TSH, sialylation of other components of thyrotropic feedback control like thyroglobulin [91] and TSH receptor [92] was observed. The effect of the above mechanisms however on overall homeostasis is still less well understood.

### 2.5. Mathematical and Simulative Models of Thyroid Homeostasis

Cybernetic models of thyrotropic feedback control help to understand the relation between structure and behaviour of the system and to predict dynamical responses to input signals and loads. Occasionally, these models may also be used as generators of hypotheses and even diagnostic procedures.

As early as 1968 Panda and Turner delivered a first quantitative description of the relation of thyroxine and TSH levels that was derived from empirical observations [115]. The first theoretically based mathematical models of thyroid homeostasis, however, had already been developed in the preceding decade [93, 94]. While these early and also some more recent models relied on a pure phenomenological approach usually on the ground of linear or polynomial relations [95, 106], improved models gradually shifted to a more and more parametrically isomorphic description, trying to map results of physiological and molecular research to a cybernetic description of the information processing structure [11, 49, 96, 97, 99, 101–103, 105, 108, 110, 111, 113] (Table 1).

As a result of increased confidence in modelling results, attempts have been made to apply some of the newer approaches [11, 23, 49, 113, 116] to clinical research [23, 117–119] and medical decision making [11, 116, 120].

**Figure 1:**
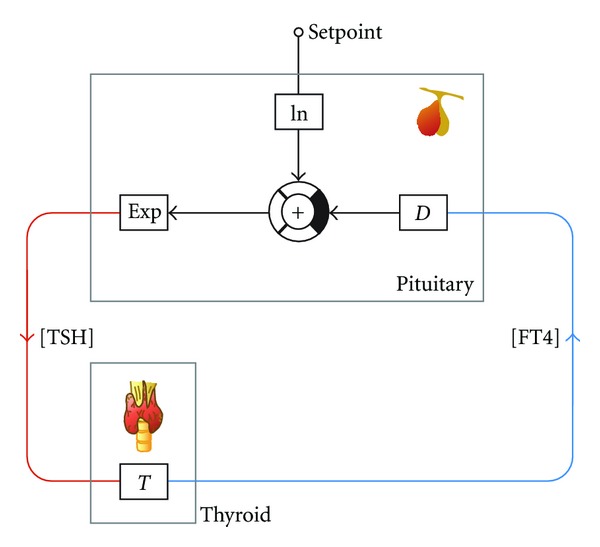
Information processing structure of the logarithmic standard model of thyroid homeostasis [105, 121].

The standard model of thyroid homeostasis (Figure 1) postulates a logarithmic relationship between FT4 levels and pituitary TSH release [105, 121]. This theory complies with empirical distributions of thyroid hormones in different populations [122, 123] and also with changes in FT3 levels in patients on substitution therapy [124]. Clinical applications try to exploit this postulated relation for diagnosis of pituitary disorders [120, 125, 126]. Recently, however, several population-based studies revealed discrepancies of bihormonal distributions from the standard logarithmic model in both euthyroidism and thyroid dysfunctions [127–129].

**Figure 2:**
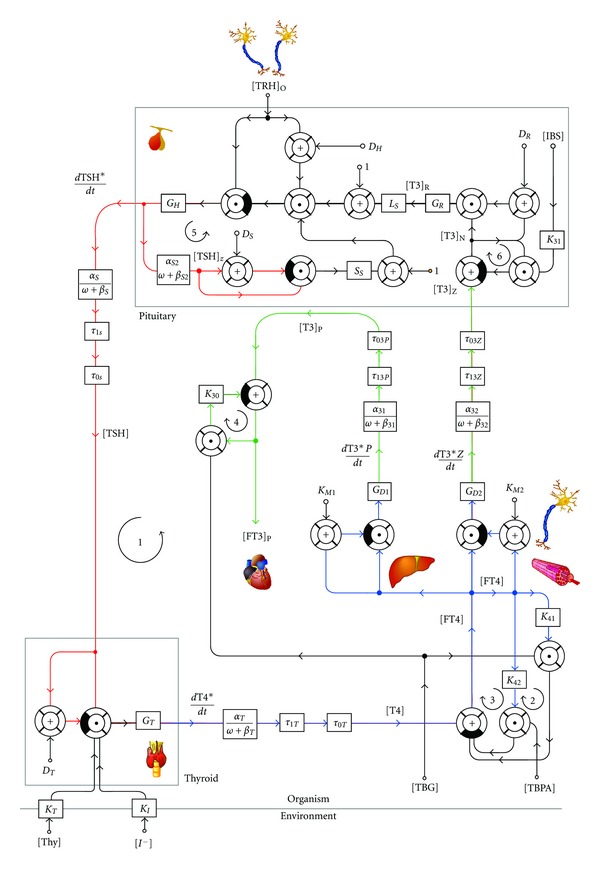
Information processing structure of a nonlinear parametrically isomorphic model based on Michaelis-Menten kinetics, noncompetitive divisive inhibition, and pharmacokinetic data [11]. Modified with permission from [49].

**Figure 3:**
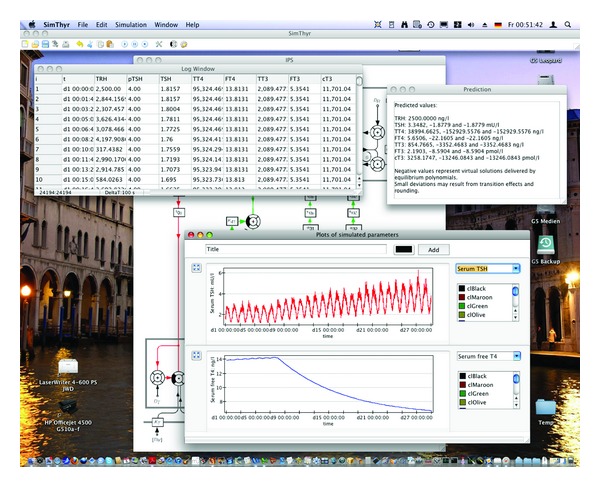
SimThyr, a continuous simulation program for thyrotropic feedback control [11].

Alternative parametrically isomorphic models (e.g., the nonlinear model depicted in Figure 2) result from “bottom-up” modelling based on molecular, cellular, and pharmacokinetic data. Not surprisingly, their mathematical theory is a great deal more complex. Therefore, computer simulations, for example, the open-source software SimThyr (Figure 3 [130]) have been developed to allow for a more intuitive understanding of the system's reactions and its temporal dynamics. The advantage of this kind of modelling approach is that parameters are well founded in thyroid biology and that resulting models therefore help to deliver hypotheses even in pathological conditions [49, 112].

“Small” models of thyroid homeostasis confine themselves to well-defined parts of the information processing structure. Examples are compartment-analytical models of iodine metabolism [131–134], kinetics of thyroid hormones [135] including their plasma protein binding [136], uptake of radioiodine [137–150], and intracellular dynamics of iodothyronine synthesis [110] and effect [151, 152].

Most models rely on parameters that have been obtained from humans. Only a minority deals with control of iodothyronine metabolism in animals, for example, cattle [133], sheep [132, 133], and rats [112].

Today, modelling of pituitary-thyroid axis is faced with the challenge of newly discovered complexities in the information processing structure, like ultrashort and long feedback loops and temporal dynamics of iodothyronine transporters. Although our knowledge benefits from excellent molecular and clinical studies, the growing intricacy of resulting models turns out to be an obstacle for continued modelling attempts.

## 3. Allostatic and Pathological Conditions

In general, pathological dysregulations may result from a disconnected feedback loop or from feed-in of autonomously generated signalling substances. The processing structure may be interrupted at virtually any site, with resulting characteristic patterns that usually allow for an estimate of where the disturbance is located.

Unlike dysregulations, allostatic responses may provide life-saving adaptation mechanisms in extreme situations, for example, in critical illness, starvation, or hibernation.

**Figure 4:**
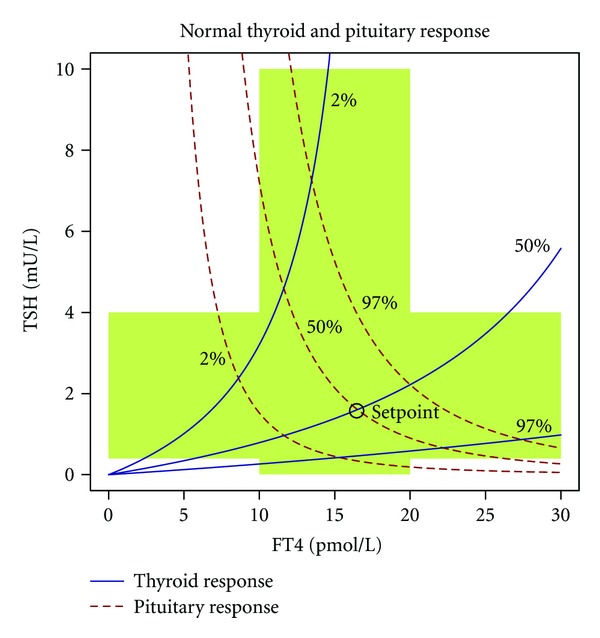
Characteristic curves of pituitary and thyroid. The area shaded in green denotes univariate reference ranges for TSH and FT4. The dashed red line denotes the pituitary's response in form of TSH incretion to varying FT4 levels; the continuous blue line represents the thyroid's response to TSH. Note that for the response curve of the thyroid—contrary to convention—the ordinate (TSH) is the independent axis, while the dependent axis is the abscissa (FT4). This uncommon notation facilitates superposition of both characteristic curves. Marked is a normal equilibrium point (also referred to as setpoint) defined by the intersection of both 50% percentiles. Response curves were calculated from percentiles for secretory capacities of pituitary (*G*_*H*_) and thyroid (*G*_*T*_) using the mathematical model displayed in Figure 2. Structure parameters were derived from a subgroup of subjects included in the NOMOTHETICOS trial [153].

In euthyroid subjects and in most cases of thyroid dysfunction the equilibrium point that is defined by steady-state levels of TSH and FT4, often in an oversimplifying manner referred to as setpoint, emanates from the intersection of characteristic curves of pituitary and thyroid (Figure 4).

Not surprisingly, the location of the equilibrium point may be modulated by changes in virtually any component of the feedback loop resulting in distortions of pituitary or thyroid characteristic curves. For instance, alterations of the setpoint have also been described in patients suffering from mutations of thyroid hormone transporters, first of all MCT8 [154–161], and in several polymorphisms of deiodinases [154, 162–167]. Additionally polymorphisms of thyroid hormone receptors [168, 169] and transcriptional cofactor heterogeneity [170, 171] may determine the location of the setpoint, but this association is still understudied.

### 3.1. Primary Thyroid Dysfunction

Primary functional disorders, marked by partial or complete disconnection of the feedback loop at the site of the thyroid, result from substantially reduced or increased thyroid's secretory capacity (*G*_*T*_), that is, the maximum stimulated amount the thyroid can produce in a given time unit. Common reasons for reduced *G*_*T*_ are autoimmune or silent thyroiditis and iatrogenic causes including thyroid surgery or radioiodine ablation. Increased *G*_*T*_ frequently ensues from heterostimulation of TSH receptors in Graves' disease or activating mutations of TSH receptors in toxic adenoma and toxic multinodular goitre [11, 116].

**Figure 5:**
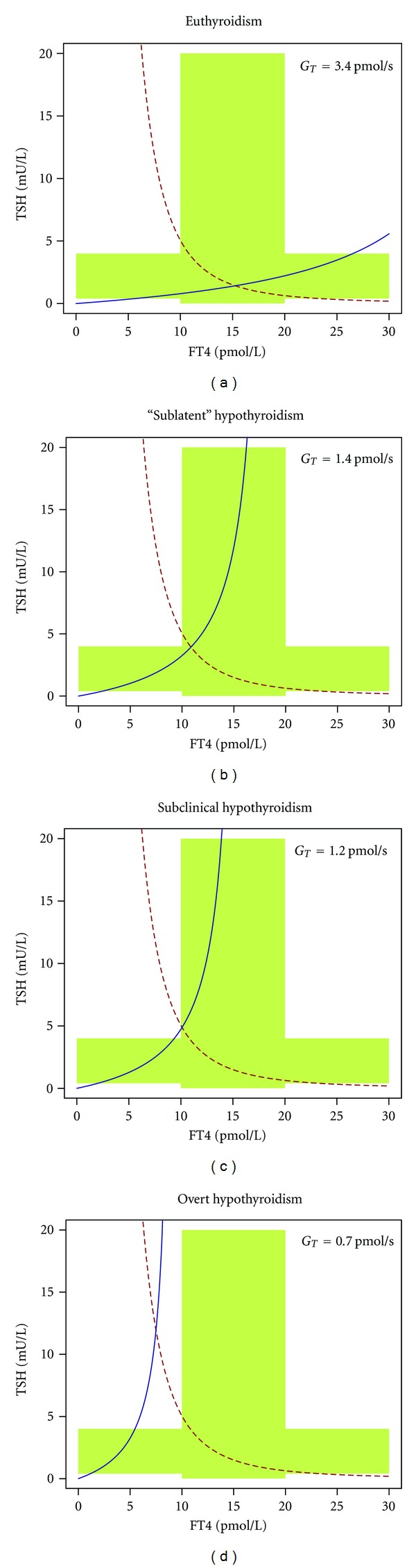
Successive development of hypothyroidism as a consequence of decreasing *G*_*T*_. Beginning with a hypothetical “sublatent” form defined by reduced *G*_*T*_ and still normal levels of TSH and FT4 (panel b), further steps are subclinical hypothyroidism with increased TSH levels and FT4 still in the lowest fraction of the reference region (panel c) and overt hypothyroidism where both parameters have left their reference region (panel d). See text for additional information.

As shown in Figure 5 the FT4 component of the equilibrium point sinks while the TSH dimension rises with decreasing *G*_*T*_. It is the nonlinear form of the response curves that gives rise to the evolution of subclinical and overt patterns of hypothyroidism.

It may astonish that the range of *G*_*T*_ resulting in subclinical hypothyroidism is rather small in the plot, although in clinical practice subclinical thyroid disorders are very common. *In vivo*, these effects may be synergistically augmented by proliferative effects of slightly reduced FT4 levels on thyrotrophs, thus effectively broadening the zone of subclinical disorders [172–174]. Other synergistic effects widening the window of subclinical hypothyroidism include long feedback and the above-mentioned alternative modes of thyroid control.

Apart from alterations in *G*_*T*_, which reflect mainly variations in the mass of functional thyroid tissue, primary heterostasis may also ensue from modified transduction properties of the TSH receptor by virtue of alterations in its dissociation constant (*D*_*T*_), for example, by nonpolymorphic mutations [175–182]. However this mechanism that underlies nonautoimmune isolated hyperthyrotropinemia is assumed to be a comparably rare condition, [183, 184]. Things are even more complex since it is expected that chronic understimulation of the thyroid by mutations in the TSH receptor eventually leads to reduced thyroid mass and thus lower *G*_*T*_ [185–187].

### 3.2. Secondary and Tertiary Thyroid Dysfunction

Central hypothyroidism or thyrotropic insufficiency, defined as reduced thyroid hormone secretion resulting from deficient stimulation of an intrinsically normal thyroid gland by TSH [188], may be of pituitary (secondary) or hypothalamic (tertiary) origin. On the basis of hormone levels and even of TRH stimulation tests, secondary and tertiary forms are nearly indistinguishable without utilization of additional information, for example, from imaging studies. To add further confusion, TSH secretion may be impaired not only quantitatively but also qualitatively resulting from secretion of biologically inactive TSH [189, 190], as discussed above.

**Figure 6:**
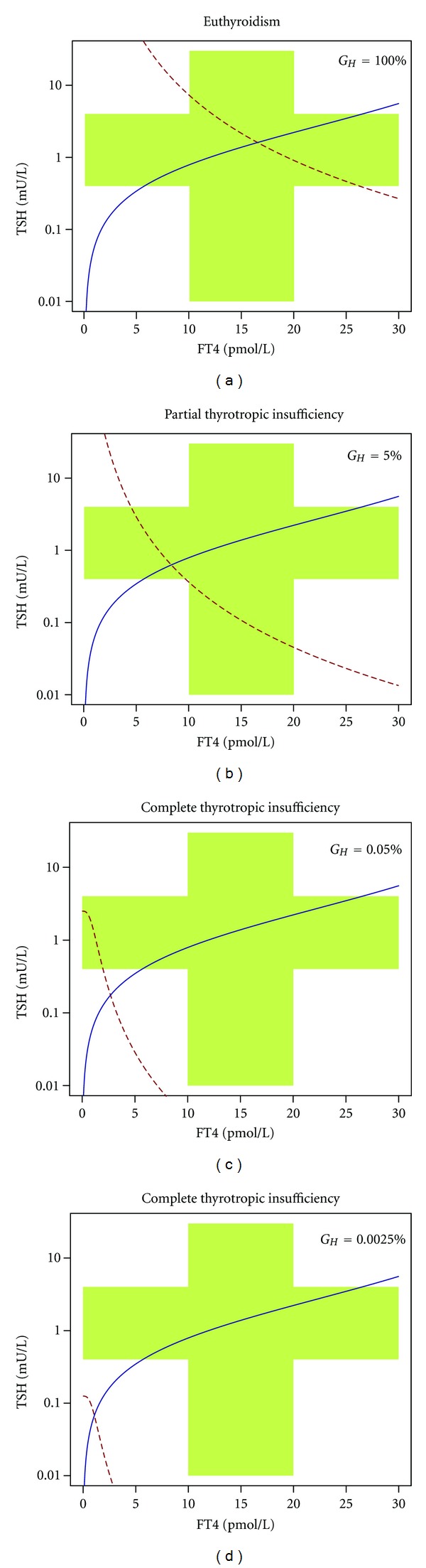
Partial and complete thyrotropic insufficiency as results of nonlinear interaction of pituitary and thyroid. TSH axis is logarithmically scaled in order to zoom small values. *G*_*H*_ values are given in percent from normal values. See text for additional information.

On a phenomenological level, however, two forms may be distinguished. In partial thyrotropic insufficiency FT4 is decreased while TSH is normal (but inadequately low in relation to reduced FT4 levels), and in complete thyrotropic insufficiency levels of both hormones are decreased. These phenotypes may be explained by nonlinear response curves of pituitary and thyroid (Figure 6).

Central hyperthyroidism, for example, resulting from TSH secreting pituitary adenomas [191] or central thyroid hormone resistance [189, 192, 193] (see Section 3.4), is a very rare condition. Interestingly, in both cases TSH levels may be excessively high, while peripheral thyroid hormone concentrations are only mildly elevated [191]. This is a consequence of nonlinear action of thyrotropin at TSHr that is well explained by the Michaelis-Menten-like input-output relation at the thyroid in some of the recent models of feedback control [11, 49].

### 3.3. Thyroid Allostasis in Critical Illness, Tumours, Uraemia, and Starvation (TACITUS)

In 1973, two independent study groups described alterations of thyroid metabolism in the starving organism [194, 195]. Later it was demonstrated that similar alterations are also common in critically ill patients and that they are associated with increased morbidity and mortality [22, 196–200].

This complex constellation, referred to as euthyroid sick syndrome or nonthyroidal illness syndrome (NTIS), is characterised by three components that may occur singly or in combination: low TSH and normal or low FT4 levels phenomenologically similar to central hypothyroidism (transient thyrotropic adaptation, occasionally leading to low-T4 syndrome) [201], impaired protein binding of thyroid hormones [202, 203], and reduced formation of T3 with simultaneously increased conversion to rT3 (low-T3 syndrome) [22–24]. Few observations report that the iodothyroacetic acids TRIAC and TETRAC are increased in NTIS and starvation [204–206]. Currently, little is known about the role of thyronamines [207] in NTIS. The fact that thyronamines are able to induce hypothermic torpor [208] and that they are a possible source of iodothyroacetate formation [209] suggests them to be increased in critical illness.

Different explanations have been proposed for the evolution NTIS. Up to now, in essence five hypotheses are discussed in the literature [22, 23].

- All observed abnormalities are the result of test artefacts by flawed assays in the presence of plasmatic interference factors. In reality, the patients are euthyroid.
- The changes in the levels of peripheral thyroid hormones mirror the effect of certain binding inhibitors that influence either laboratory determinations only or also the transfer of thyroid hormones into tissue of diseased persons and thus diminish binding of iodothyronines to T3 receptors.
- Due to increased local deiodination, T3 levels are normal in the pituitary gland while they are low in the rest of the organism.
- Levels of peripheral thyroid hormones are actually low so that affected patients are biochemically hypothyroid. However, this useful physiological function should not be interfered with.
- NTIS is a form of central hypothyroidism. The resulting tissue hypothyroidism should be treated with appropriate substitution therapy.

Today, NTIS is still poorly understood from an integrative view. As similar alterations are observed with very different assay techniques and a comparable phenotype is also observed in starving or hibernating organisms [210–214] it seems to be more than only the result of flawed assays. Obviously, it is an extreme form of a more general allostatic response ensuring survival in certain stress situations. We therefore propose the more neutral term *thyroid allostasis in critical illness, tumours, uraemia, and starvation* (TACITUS) for this form of adaptation taking place in a broader context of physiological extremes. 

Provided that patients with NTIS are faced with poor prognosis, several trials have been conducted to evaluate the question of a possible treatment [22, 197]. However, their results were ambiguous. Some studies could show a benefit of substitution therapy with thyroid hormones, for example, regarding the incidence of atrial fibrillation [22, 197, 215, 216] and hemodynamic parameters [217, 218] while others could not observe relevant differences in outcome between treated and untreated patients [219–221] or even described detrimental effects of substitution therapy [222–225].

**Figure 7:**
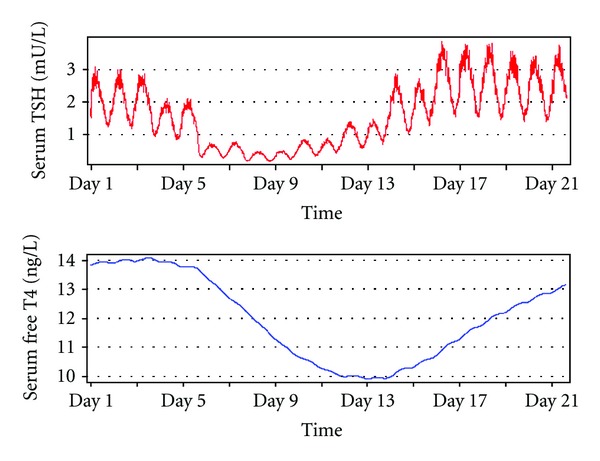
Computer simulation of thyrotropic adaptation in critical illness. A gradual increase of central type 2 deiodinase activity over several days with subsequent restoration to normal values has been simulated with SimThyr using the mathematical model shown in Figure 2. Note the temporarily increased TSH values after day 17 that are occasionally observed also *in vivo* in patients recovering from nonthyroidal illness syndrome.

Today, more and more investigations reveal a fundamental, albeit not exclusive, role of central components in the evolution of TACITUS [22–24, 61, 226]. This may even apply to hypodeiodination leading to low-T3 syndrome, as TSH is able to stimulate *D*2 expression via cAMP [26, 227–230]. Conversely, central hyperdeiodination may lead to suppressed TSH levels, as both evidenced by animal experiments [61, 231] and computer simulations (Figure 7).

Thyrotropic adaptation is a challenge affecting clinical practice, as it is nearly indistinguishable from latent (subclinical) hyperthyroidism—although pathophysiology and therapeutic implications are opposed [196, 232].

### 3.4. Rare Conditions of Thyroid Function

In most cases high-T3 syndrome and T3 thyrotoxicosis result from T4 hyperthyroidism with consecutive surplus substrate supply for deiodinases. Isolated high-T3 syndrome with normal or even low FT4 levels is a rare form of NTIS that is caused by hyperdeiodination. Cases of high-T3 syndrome have been described in toxic adenoma [233], Graves' disease [234], nodular goitre [233], follicular thyroid carcinoma [235, 236], and systemic sclerosis [237]. Increased TSH signalling in the first two conditions gives further evidence for the relevant role of thyrotropin in control of deiodinase activity [238].

Thyroid hormone resistance is usually caused by mutation of the nuclear thyroid hormone receptor beta gene (TR-beta), with a resulting hormone pattern similar to central hyperthyroidism and a split phenotype of clinical thyrotoxicosis with regard to peripheral organs and heterogeneous manifestations at the site of the central nervous system [192, 239].

Recently, the first case of thyroid hormone receptor alpha mutation was reported. The phenotypical pattern consisted in skeletal abnormalities, microsomia, constipation, and hyperdeiodination [240].

The existence of acquired partial thyroid hormone resistance has been postulated [241, 242], but this condition may be rare or underrecognised. In NTIS, however, disruption of thyroid hormone signalling by cytokines, metabolites, toxins or drugs may contribute substantially to the clinical phenotype of affected patients [242]. Possible mechanisms of acquired thyroid hormone resistance include impairments of transmembrane transport [15, 16, 243], deiodination [19, 227, 228, 244], entry into nucleus [245, 246], receptor binding [243, 247–253], and nongenomic effects of iodothyronines [28, 254–264]. Similar effects may ensue from exposure to endocrine disruptors [265] like phthalates [266–268], brominated flame retardants [266, 267, 269], perfluorinated compound [266, 267, 270], polychlorinated biphenyls [271–276], bisphenol A [254, 266–269, 274], or bisphenol F [277].

Some environmental toxins may also act as thyroid hormone agonists, as demonstrated for certain polychlorinated biphenyls [278, 279].

### 3.5. Calculated Structure Parameters as Diagnostic Methods Beyond Univariate Hormone Determinations

Decision making based on TSH levels alone may lead to misinterpretations of serious impact, especially in cases of possible overt thyroid heterostasis [127–129] and even more in potential central dysregulations [280]. However, introducing FT3 or FT4 levels while leaving the process of diagnostic reasoning in a univariate manner does not prove to be helpful due to the lack of combination rules and low diagnostic sensitivity of peripheral thyroid hormone levels [11].

Combining hormone levels with model-based calculations delivers structure parameters of thyroid homeostasis that may in certain conditions add valuable information for clinical research and differential diagnosis of thyroid disorders, even beyond of classical primary and secondary heterostasis.

The simplest and probably earliest method to exploit existing knowledge about thyroid homeostasis for diagnostic purposes is calculating T3/T4 ratio. It can be calculated from either total or free thyroid hormones. T3/T4 ratio has been applied in numerous publications, and it was shown that this parameter is elevated in certain thyroid disorders [234, 236, 281, 282] and iodine deficiency [283], while it is reduced in nonthyroidal illness [284, 285]. Furthermore, T3/T4 ratio mirrors nutritional [286] and drug effects on deiodination [287], and it may be useful to distinguish thyroiditis from other causes of thyrotoxicosis [288]. Reduced T3/T4 ratio in central hypothyroidism is another hint for the stimulating role of TSH for deiodination [289].

The T3/T4 ratio ignores fundamental biochemical principles by implying a linear relationship between T3 and T4. This is corrected by an alternative approach, calculating sum activity of peripheral deiodinases (*G*_*D*_, also referred to as SPINA-*G*_*D*_) with

(1) $$\begin{array}{c} {\hat{G}}_{D}=\frac{\beta_{31}\left(K_{M1}+\left[\text{FT}4\right]\right)\left(1+K_{30}\left[\text{TBG}\right]\right)\left[\text{FT}3\right]}{\alpha_{31}\left[\text{FT}4\right]} \end{array}$$

from free T4, free T3, and parameters for protein binding, dissociation, and hormone kinetics (Table 2) [11, 23]. The equation had been derived from a nonlinear model of thyrotropic feedback control by solving the transfer equation for *G*_*D*_ under the condition of equilibrium [49]. It could be demonstrated that *G*_*D*_ correlates with body mass index [11]. Additionally, the parameter was observed to be reduced in certain forms of NTIS including renal failure [290] and inflammatory bowel diseases [291].

The T3/rT3 ratio is a measure of relative contributions of type 2 and type 3 deiodinases on deiodination of T4. It was observed to be decreased in critical illness [296, 297] and hyperthyroidism [298], and to be increased in insulin resistance [299].

Theoretical thyroid's secretory capacity (*G*_*T*_ or SPINA-*G*_*T*_) denotes the maximum amount of thyroxine the thyroid can produce in a given time unit under stimulated conditions. Formally similar to *G*_*D*_, it can be calculated *in vivo* with

(2) $$\begin{array}{c} {\hat{G}}_{T}=\frac{\beta_{T}\left(D_{T}+\left[\text{TSH}\right]\right)\left(1+K_{41}\left[\text{TBG}\right]+K_{42}\left[TBPA\right]\right)\left[\text{FT}4\right]}{\alpha_{T}\left[\text{TSH}\right]} \end{array}$$

from TSH levels, FT4 levels, and constant parameters for kinetics and protein binding (Table 2) [23]. *G*_*T*_ has been observed to correlate with thyroid volume as determined by ultrasonography and to be elevated in hyperthyroidism and reduced in hypothyroidism [11, 116]. Furthermore, *G*_*T*_ correlates with creatinine clearance suggesting a negative influence of uremic toxins on thyroid biology [290]. In healthy volunteers *G*_*T*_ showed a higher reliability compared with TSH, FT4, or FT3 (Table 3) [11]. These results imply that, unlike *G*_*D*_ or univariate hormone levels that mirror acute regulation, *G*_*T*_ might represent a constant parameter of thyroid homeostasis.

Recently, a small study that has been published as abstract revealed calculating *G*_*T*_ to be beneficial in differential diagnosis of NTIS with thyrotropic adaptation and latent (subclinical) hyperthyroidism [301].

Additionally, specific thyroid's secretory capacity (*G*_*TS*_) had been defined by calculating a ratio of *G*_*T*_ and thyroid volume, as determined, for example, by ultrasonography [11]. This structure parameter denotes the maximum amount of thyroxine that can be produced by 1 mL of thyroid tissue under stimulated conditions. In one study a significant positive correlation between body mass or BMI, respectively, and thyroid volume was observed, while in the same population the correlation between body mass and *G*_*TS*_ was negative, suggesting reduced functional quality of thyroid tissue in obesity [11].

Thyrotroph T4 Sensitivity Index (TTSI)

(3) $$\begin{array}{c} \text{TTSI}=\frac{100\left[\text{TSH}\right]\left[\text{FT}4\right]}{l_{u}} \end{array}$$

with *l*_*u*_ being the upper limit of the reference interval for FT4 has been suggested as a screening parameter for thyroid hormone resistance [302]. However up to now, this parameter has not been widely adopted. TSH-FT4 product, a similar measure, was demonstrated to have a significant heritable component in a large cohort of twin pairs [303]. 

An alternative method to assess thyrotropic function of anterior pituitary is Jostel's TSH index [120]. This calculated parameter is based on the logarithmic standard model of thyroid homeostasis. Calculating

(4) $$\begin{array}{c} \text{TSHI}=\ln\;\left[\text{TSH}\right]-\beta \left[\text{FT}4\right] \end{array}$$

delivers a raw value. A second standardised form of TSHI is based on mean values (2.7) and standard deviations (0.676) of TSHI

(5) $$\begin{array}{c} \text{sTSHI}=\frac{\text{TSHI}-2.7}{0.676}. \end{array}$$

TSHI predicts the risk of failure in dynamic pituitary testing and correlates with functional measures of other anterior pituitary axes [120].

Although calculated structure parameters may add value to the determination of classical univariate hormone values, their informative value depends in a critical manner on the quality of the assays used for underlying hormone measurements. Although assays for TSH, free, and total peripheral thyroid hormones have been continually improved over the previous decades [304–312], some indeterminacy persists [307, 313–315]. Additionally, if certain input parameters, for example, TBG and binding constants for calculation of *G*_*T*_ and *G*_*D*_, are not biochemically determined but, as usual, replaced by standard values, some bias may ensue. Therefore, *G*_*T*_ and *G*_*D*_ will be overestimated in NTIS, where plasma protein levels are reduced [202, 203]. Although this will usually not pose problems in differential diagnosis, as the impairment of protein binding affects all investigated groups, the person calculating structure parameters and interpreting their results should at least be aware of these difficulties. Of course, the same considerations also apply to empirical parameters like TTSI and TSHI, the more, as here the origin of bias is less obvious, since protein levels and binding constants are not explicitly stated in the equations.

## 4. Alternative Thyrotropic Agonists

TSH is one of five related glycoprotein hormones consisting of two noncovalently bound chains. TSH, LH, FSH, and HCG share a common alpha subunit that is encoded on human chromosome 6 and contains a protein core of 92 amino acid residues in humans [316, 317]. The specific information is encoded by the beta subunit that has a different amino acid sequence for each hormone, and especially a certain “seat belt” region, where the beta chain wraps around the alpha chain [318, 319]. Free alpha or beta subunits are devoid of bioactivity. A fifth glycoprotein hormone, thyrostimulin (TSH 2) with a similar molecular structure, has been described. It contains both a different alpha and beta subunit [320]. 

Gene expression of both alpha and beta subunits are controlled positively by a PKA/PKC-CBP-CREB pathway that is stimulated by TRH and AVP and inhibited by Dopamine. Additionally, expression of both subunits is inhibited by a negative thyroid hormone response element (nTRE) that is dependent on TR-beta receptor [316] signalling.

**Figure 8:**
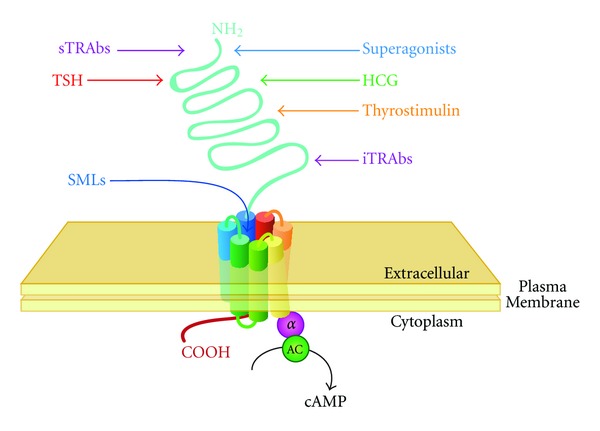
Interaction of TSH, thyrotropic agonists, and thyrotropic antagonists with TSH receptor. SMLs bind to a pocket within the heptahelical transmembrane domain, while TSH, HCG, and TRAbs bind primarily to the TSHr amino-terminal ectodomain.

TSH and thyrotropic agonists bind to the TSH receptor (TSHr), a heptahelical G-protein-coupled receptor that has homologies to FSHr and LH/CGr [321] (Figure 8). Like in TSH (see Section 2.4), variable sialylation of glycosylated side-chains modifies bioactivity of glycoprotein hormones [81, 85, 91, 322], for example, by affecting cooperative effects of individual TSH domains in receptor activation [317].

Due to the above-mentioned considerations it is not surprising that considerable crosstalk exists between thyrotropic and gonadotropic feedback controls. In addition, TSHr antibodies [323] as well as small molecule “drug-like” ligands [324, 325] may stimulate or block signal transduction.

### 4.1. HCG

Although in human embryos the thyroid is able to produce T3 and T4 in the 10th or 11th week, and pituitary thyrotrophs are detectable in the 13th week, maturation of functional thyrotropic feedback control is not effective before the 18th to 20th week [326, 327].

Therefore, the embryo is dependent from maternal supply with thyroid hormones in the first half of pregnancy. This and the fact that the binding of iodothyronines to TBG is increased in pregnancy require some upregulation of T4 biosynthesis.

Sequence similarities between TSH and HCG, and between their receptors, allow for some promiscuous activation of TSHr by HCG in the first trimester of pregnancy [321].

Stimulation of the thyroid gland by HCG in pregnancy accounts for an inverse relationship between serum concentrations of TSH and HCG [328]. In cases of extremely elevated HCG levels, for example, in hydatidiform mole or chorionic carcinoma, overt hyperthyroidism and even thyroid storm may ensue [329–331]. The same pathomechanism may lead to hyperthyroidism in embryonal testicular carcinoma [332, 333].

Interestingly, heterostimulation of TSHr by HCG is a matter of both quantity and quality. In Chinese hamster ovary cells transfected with human TSHr enhanced thyrotropic activity was observed in sera from women with trophoblastic disease. If CHO cells were transfected with LH/CGr, however, cAMP production was higher in sera from women with normal pregnancy. These diverging results may ensue from microheterogeneity of hCG, for example, in form of different carbohydrate modifications [334, 335].

Mutations of TSH receptor leading to increased sensitivity to HCG may lead to overt hyperthyroidism even in normal pregnancy [336].

Theoretically, HCG should also be able to activate pituitary TSH receptors and lead to suppression of TSH secretion via the Brokken-Wiersinga-Prummel loop. Although this possibility could be useful for targeted therapy of hypothyroidism in pregnancy, up to now no data are available from clinical trials.

### 4.2. Thyrostimulin

Based on GenBank searches Nakabayashi et al. identified two additional human glycoprotein hormone subunit-like genes with structural similarity to the genes of the common alpha subunit and the beta chain for TSH, respectively. Using a yeast two-hybrid assay they found the two units to be able to heterodimerize and finally they confirmed their colocalization in the anterior pituitary and the ability of the resulting heterodimeric protein to bind and activate human TSH receptors, but not LH and FSH receptors [320]. Consequently, they named the A2/B5 heterodimeric glycoprotein thyrostimulin; other designations are TSH 2 or corticotroph-derived glycoprotein hormone (CGH), as both chains are expressed in corticotrophs of anterior pituitary [337].

Today, the physiological role of thyrostimulin is still not well understood. It has been proposed to play a role for Brokken-Wiersinga-Prummel loop [338] or in paracrine effects within the pituitary [320]. It has also been hypothesised that thyrostimulin may be responsible for thyroid heterostimulation in cases of diffuse thyroid autonomy [339], but this assumption has not been proved up to now. Expression of thyrostimulin subunits was observed in pituitary, central nervous system, adrenal gland, gastrointestinal organs, retina, skin, and testes [340]. LPS and inflammatory cytokines are able to upregulate thyrostimulin expression [338, 341], which may be another factor in the pathogenesis of thyrotropic adaptation in NTIS.

### 4.3. Thyrotropin Receptor Antibodies

Classical Graves' disease (autoimmune thyroiditis type 3A) is caused by formation of stimulating TSH receptor antibodies (sTRAbs, also referred to as thyroid stimulating antibodies, TSAbs) by intrathyroidal B cells with resulting hyperthyroidism. Retroorbital TRAb formation leads to endocrine ophthalmopathy, and it is assumed that other manifestations of Graves' disease derive from extrathyroidal TRAb effects, too.

As mentioned above sTRAbs may suppress TSH secretion independently from FT4 levels by activation of the Brokken-Wiersinga-Prummel loop [47]. Therefore, in Graves' disease low TSH levels may persist despite even low FT4 levels [50]. Obviously, this also applies to children born to mothers with Graves's disease and high antibody load [41]. Immunogenic TSH suppression may complicate diagnosis of thyroid status and dosage of thyroid hormones or thyrostatic agents in Graves' disease.

Inhibiting TSH receptor autoantibodies (iTRAbs, or TSH-stimulation blocking antibodies, TSBAbs) block signal transduction at the TSH receptor. Up to now, their effect on global thyroid homeostasis or ultrashort loop control of TSH secretion has not been investigated, which may also be a consequence of still limited availability of reliable sTRAb and iTRAb assays for routine use. Over the time, the proportion of stimulating or inhibiting TRAbs may change in individual patients [342].

Synthetic TSH receptor antibodies are described below.

### 4.4. Orosomucoid

Orosomucoid, also referred to as alpha-1-acid glycoprotein (AGP), is an acute-phase glycoprotein that is synthesised primarily in hepatocytes. It is known to act as a carrier of neutrally charged and basic lipophilic molecules [343, 344]. AGP in low concentrations was observed to stimulate the TSH receptor and intracellular cAMP accumulation. On the other hand, high concentrations of AGP inhibited TSH signalling [345]. Orosomucoid might therefore play a role in the pathogenesis of NTIS, but additional studies are needed to get a more thorough understanding of its role in thyroid physiology.

### 4.5. Synthetic TSH Receptor Agonists and Antagonists

Recently, a wide range of substances stimulating or blocking signal transduction at the TSH receptor has been developed. These agents may be divided in agonists (that activate receptors), neutral antagonists (that inhibit receptor activation by agonists, but do not display any activity on their own), and inverse agonists (that both block receptor activation by agonists and inhibit basal, constitutive signalling in an agonist-independent manner). TSHr ligands have been isolated in form of monoclonal antibodies, engineered glycoprotein hormones, and small molecules.

The first attempts to obtain monoclonal TSH receptor antibodies resulted in low-affinity and partly low-specific MAbs [346–354]. In the past decade, however, different high-affinity monoclonal TRAbs with agonist activity (TSMAbs 1–3 [355], MS-1 [356], M22 [357], and K1-18 [358]), neutral antagonist activity (Mab-B2 [359] and k1-70 [358]), and inverse agonist activity (5C9 [360, 361]) have been isolated [323, 362, 363]. These macromolecules helped to understand important pathophysiological aspects of Graves' disease and to develop and validate TRAb test kits [364].

A series of superactive analogues of mammalian glycoprotein hormones has been designed by a combination of evolutionary mechanisms, sequence comparisons, and homology modelling. The resulting superagonists demonstrated substantial increases in receptor binding affinity and intrinsic activity [365, 366]. Similarly, increased bioactivity was observed in a construct of TSH alpha and beta chains fusioned to a single polypeptide [367].

Small molecule “drug-like” ligands (SMLs) exhibit different binding properties. Similar to MAbs, SMLs have been developed with agonistic (NCGC00161870), neutral antagonistic (NCGC00242595), and inverse agonistic activity (NCGC00161856, NCGC00229600, and Org 274179-0) [324, 325], but unlike antibodies they bind to a pocket within the transmembrane domain (Figure 8), thus also being able to activate TSH receptors bearing mutations in their ectodomain.

Agonistic TSHr ligands may be useful thyroid stimulators in patients with thyroid cancer in place of rhTSH for radioiodine therapy, thyroid scan, or thyroglobulin determination. Neutral antagonists could be beneficial for patients suffering from Graves' disease or endocrine ophthalmopathy, and inverse agonists may be a perspective in the treatment of toxic adenoma or thyroid cancer, in the latter case as an adjunct or substitute to TSH suppression [323, 324].

## 5. The TSH Reference Range—An Ongoing Controversy

Measuring serum levels of TSH and total or free peripheral thyroid hormones delivers univariate reference ranges that are usually defined by a tolerance region covering 95% of healthy individuals.

Recently, a new debate on the boundaries of TSH reference range has emerged [368, 369], since it was observed that patients with TSH levels of more than 2.5 mU/L are exposed to increased risk of developing overt hypothyroidism [370–374]. Additionally, in this group of what we would suggest to term “sublatent hypothyroidism,” elevated levels of thyroid autoantibodies [375, 376] and increased frequency of hypoechogenicity in thyroid ultrasound [377] were observed. Moreover, there is evidence that the intraindividual variation of TSH levels is narrower than the width of the population based reference range [378, 379].

These findings may result from the effect of thyrotropic feedback control in general and from nonlinear distortions of the FT4-TSH relationships in euthyroid individuals and patients with thyroid dysfunction [127–129].

Therefore, it has been postulated to lower the upper limit of the TSH reference range from 4 mU/L to 2.5 mU/L [380–382]. However, this suggestion is subject to disputation, as lowering the reference range border would lead to a high number of false positive results and an increase in health-care expenses [383].

Alternative biomarkers to assess the supply of the organism with thyroid hormones include resting heart rate and other determinants of cardiac output [384, 385], oxygen consumption [386, 387], respiratory quotient [388, 389], thermogenesis [26, 390–392], methylhistidine excretion [393–397] and plasma levels of lipids [398, 399], SHBG [400–405], sclerostin [406], ceruloplasmin [407], lead [408], copper [407–410], arsenic [408], or MBL [411, 412]. These parameters have not been well evaluated, however, and due to the fact that they are subject to multiple extrathyroidal influencing factors they are not expected to deliver superior results as diagnostic tools.

**Figure 9:**
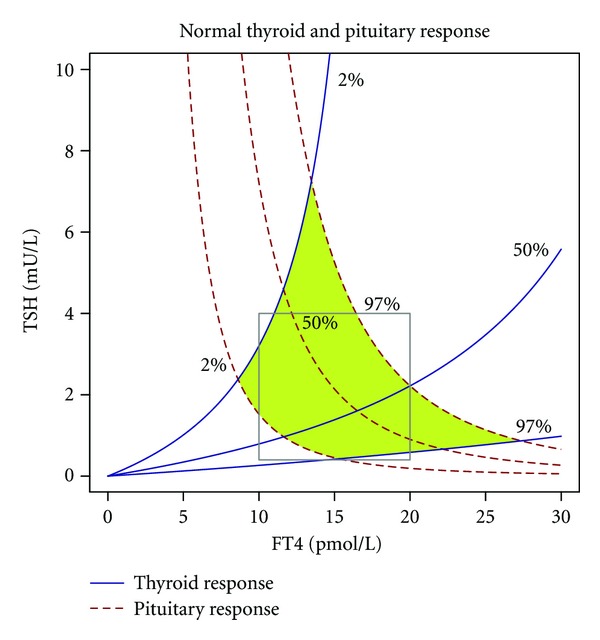
Comparison of conventional univariate reference ranges for TSH and FT4 (grey box) and a bihormonal reference region (green kite-like area) from nonlinear modelling of thyroid homeostasis. For more information see text and legend of Figure 4.

A possible solution could be interpretation of laboratory results based on a system's level understanding of thyrotropic feedback control. As shown in Figure 9 a complex reference region based on percentiles of pituitary and thyroid response has considerable overlap with conventional univariate reference values, but also significant deviations in the corners of the respective regions. As a result, a TSH level of 6 mU/L may be normal if FT4 level is 13 pmol/L, but a sign of primary hypothyroidism if FT4 level is 10 pmol/L. Conversely, a TSH level of 0.5 mU/L would suggest central hypothyroidism if FT4 is 11 pmol/L, but be normal if FT4 is 16 pmol/L.

Today, this is not more than a perspective for a more differentiated method to-be for interpreting results of thyroid hormone determinations. Future studies evaluating this approach in clinical settings are warranted.

Another challenge, especially for dosage of substitution therapy with levothyroxine, is the fact that the two-dimensional location of the individual equilibrium point (setpoint) is unknown in the targetpopulation. Obviously, the individual setpoint would be the ideal target for dosing algorithms, but unfortunately, in clinical practice it is impossible to infer its location from characteristic curves of pituitary and thyroid gland, as the thyroid response curve is either distorted or vanished in thyroid disorders. It is another task for future clinical research to find a methodology to reconstruct the setpoint from pituitary behaviour or metabolic markers of thyroid signalling.

Lastly, substitution therapy may be complicated by interindividual variations of deiodination. The question, if monotherapy with T4 or a combination of T4 and T3 should be preferred, is for years subject of debate. Numerous trials [413–422] did not lead to a standard recommendation. Persons with abnormal sum activity of deiodinases, however, might benefit from additional treatment with liothyronine [414, 423], although this does not hold true for all polymorphisms of deiodinases [424]. Calculating *G*_*D*_ or T3/T4 ratio might help to stratify patients for an individualised therapy, but the required trials are still to be designed.

## 6. Conclusions

Methodological advances in mathematical and simulative modelling of thyroid homeostasis have led to a better understanding of static and dynamic behaviour of thyroid hormones in health and disease. Together with results from molecular and clinical research on the central role of TSH in thyroid homeostasis such progress has permitted the development of advanced methods for interpretation of laboratory results that provide previously inaccessible information on pituitary and thyroid function. A future perspective overcoming the limits of univariate reference ranges for TSH, FT4, and FT3 promises the development of approaches for personalised diagnosis of thyroid homeostasis that may also be a foundation for targeted dosing of thyroid hormone substitution.

## Figures and Tables

**Table 1:** Overview of published models of thyrotropic feedback control. Applications for research refer to any scientific exploitation outside of the modelling context itself, for example, for reasoning in clinical trials or generation of hypotheses.

Authors	Year	Transfer characteristics	Type of modelling approach	Applications for research	Clinical applications	Reference
Danziger and Elmergreen	1956	Linear	Phenomenological	−	−	[93]
Roston	1959	Linear with basal secretion	Phenomenological	−	−	[94]
Norwich and Reiter	1965	Linear	Phenomenological	−	−	[95]
DiStefano and Stear	1968	Linear with basal secretion	Phenomenological, partly parametrically isomorphic	−	−	[96]
DiStefano and Chang	1969, 1971	Linear with basal secretion	Phenomenological, partly parametrically isomorphic	−	−	[97, 98]
DiStefano et al.	1975	N/A	Parametrically isomorphic	−	−	[99]
Sudova and Langer	1975	Exponential with compartment-analytical components	Phenomenological, partly parametrically isomorphic	−	−	[100]
Saratchandran et al.	1976	Logarithmic and linear	Phenomenological, partly parametrically isomorphic	−	−	[101]
Seif	1977	Logarithmic and linear	Phenomenological, partly parametrically isomorphic	−	−	[102]
Wilkin et al.	1977	Limit elements	Phenomenological, partly parametrically isomorphic	−	−	[103]
Hatakeyama and Yagi	1985	Linear with first order time constants	Phenomenological	−	−	[104]
Cohen	1990	Logarithmic	Phenomenological	+	+	[105]
Li et al.	1995, 1994	Complex polynoms	Phenomenological, partly parametrically isomorphic	−	−	[106, 107]
Dietrich et al.	1997	Linear and Michaelis-Menten kinetics	Phenomenological, partly parametrically isomorphic	−	−	[108]
Dietrich et al.	2002, 2004	Michaelis-Menten kinetics, noncompetitive divisive inhibition, first order time constants	Parametrically isomorphic (parameters for adult humans)	+	+	[11, 49]
Falaschi et al.	2004	Linear	Phenomenological	−	−	[109]
Degon et al.	2008	Based on compartment and flux analysis	Phenomenological, partly parametrically isomorphic	−	−	[110]
Leow	2007	2nd order Bernoulli differential equations, hysteresis, inverse exponential power law of TSH response	Phenomenological, partly parametrically isomorphic	−	−	[111]
Mclanahan et al.	2008	Michaelis-Menten kinetics, noncompetitive divisive inhibition, first order time constants	Parametrically isomorphic (parameters for adult rats)	−	−	[112]
Eisenberg et al.	2008, 2010	Based on earlier models by DiSefano et al.	Phenomenological, partly parametrically isomorphic	+	−	[113, 114]

**Table 2:** Constant parameters for diagnostic calculations.

Symbol	Explanation	Value	Reference
*β*	Correction coefficient for log-linear model	−0.1345	[120]
*α*_*T*_	Dilution factor for T4 (reciprocal of apparent volume of distribution)	0,1 L^−1^	[11, 23]
*β*_*T*_	Clearance exponent for T4	1,1 ∗ 10^−6^ sec^−1^	[11, 23, 292]
*D*_*T*_	EC_50_ for TSH	2,75 mU/L	[11, 23, 293]
*K*_41_	Dissociation constant T4-TBG	2 ∗ 10^10^ L/mol	[11, 23, 106]
*K*_42_	Dissociation constant T4-TBPA	2 ∗ 10^8^ L/mol	[11, 23, 106]
*α*_31_	Dilution factor for T3	0,026 L^−1^	[11, 23]
*β*_31_	Clearance exponent for T3	8 ∗ 10^−6^ sec^−1^	[11, 23]
*K*_*M*1_	Dissociation constant of type 1 deiodinase	5 ∗ 10^−7^ mol/L	[11, 23, 294]
*K*_30_	Dissociation constant T3-TBG	2 ∗ 10^9^ L/mol	[11, 23, 295]

**Table 3:** Test-retest reliability measures of TSH, FT4, FT3, SPINA-GT, and SPINA-GD from repeated measurements with at least one month interval in 20 healthy volunteers from the SPINA network [11, 116]. *e*: repeatability = (interindividual variance)/(intraindividual variance + interindividual variance) [300]. Larger figures denote higher reliability.

Parameter	*e*	*R* ^2^
TSH	0.63	0.16
FT4	0.71	0.35**
FT3	0.68	0.36**
SPINA-GT	0.73	0.42**
SPINA-GD	0.64	0.36**

**P < 0.01.

## Abbreviations

AGP: Alpha-1-acid glycoprotein (orosomucoid)
AVP: Arginine-vasopressin
cAMP: Cyclic adenosine monophosphate
CBP: CREB-binding protein
CREB: cAMP response element
FSH: Follicle-stimulating hormone
FSHr: FSH receptor
FT3: Free triiodothyronine
FT4: Free thyroxine
*G*_*D*_: “Gain of deiodinase,” sum activity of activating deiodinases
*G*_*T*_: “Gain of thyroid,” secretory capacity of the thyroid gland
HCG: Human chorionic gonadotropin
iTRAb: Inhibiting TSH receptor autoantibody
LH: Luteinizing hormone
LH/CGr: Receptor for LH and HCG
MAb: Monoclonal antibody
MBL: Complement mannan-binding lectin
NTIS: Nonthyroidal illness syndrome
PKA: Protein kinase A
PKC: Protein kinase C
rhTSH: Recombinant human TSH
rT3: Reverse triiodothyronine
SHBG: Sex hormone binding globulin
SMLs: Small molecule “drug-like” ligands
sTRAb: Stimulating TSH receptor autoantibody
sTSHI: Standardised TSH index
T3: Triiodothyronine
T4: Thyroxine
TACITUS: Thyroid allostasis in critical illness, tumours, uraemia and starvation
TBPA: Transthyretin
TRAb: TSH receptor autoantibody
TRH: TSH releasing hormone, thyroliberin
TSAb: Thyroid stimulating antibody
TSBAb: TSH-stimulation blocking antibody
TSH: Thyroid stimulating hormone, thyrotropin
TSH 2: thyrostimulin
TSHI: Jostel's TSH index
TSHr: TSH receptor.
